# Bullous Pemphigoid Associated With Pembrolizumab Therapy for Non-Small-Cell Lung Cancer: A Case Report

**DOI:** 10.7759/cureus.21770

**Published:** 2022-01-31

**Authors:** Tulika Chatterjee, Thomas F Rashid, Salman B Syed, Moni Roy

**Affiliations:** 1 Internal Medicine, University of Illinois College of Medicine at Peoria, Peoria, USA

**Keywords:** immune checkpoint inhibitor adverse effects, pembrolizumab cutaneous side effect, immune checkpoint inhibitor, pembrolizumab, bullous pemphigoid

## Abstract

Pembrolizumab is an immune checkpoint inhibitor being increasingly used as immunotherapy for a multitude of cancers. With the increasing use of these agents, various immune-related adverse events are being recognized. Lichenoid reaction, pruritus, and eczema are well-established cutaneous side effects of pembrolizumab, but bullous pemphigoid (BP) is a rare side effect of the drug. It is difficult to establish this diagnosis because it needs a detailed history of the timeline of the evolution of symptoms and careful ruling out of other etiologies, especially other drugs. Here, we present the case of a 66-year-old male who developed BP after receiving pembrolizumab therapy for non-small-cell lung cancer. Discontinuation of pembrolizumab and the use of topical and systemic steroids led to the complete resolution of symptoms. Physicians should be aware of this potential complication and keep it on their differential diagnosis when treating patients on immune checkpoint inhibitors.

## Introduction

Bullous pemphigoid (BP) is the most common pemphigoid disorder with autoantibodies against dystonin leading to dermal-epidermal lysis [1,2]. Several medications have been identified to precipitate BP. Pembrolizumab is an immune checkpoint inhibitor targeting programmed cell death protein-1 (PD-1) receptors on lymphocytes. In recent years, there have been expanding indications for the use of pembrolizumab for the treatment of various malignancies. It is important to be aware of immune-related adverse events (irAEs) that are commonly associated with checkpoint inhibitors. The irAEs that have been associated with pembrolizumab include hypothyroidism/hyperthyroidism, colitis, hepatitis, pneumonitis, nephritis, and various cutaneous events [3]. The most common cutaneous adverse events associated with pembrolizumab include lichenoid reactions, eczema, and vitiligo [4]. Other adverse cutaneous events such as pruritus, seborrheic keratoses, and bullous pemphigoid have also been reported [4]. BP due to pembrolizumab is not very common but has been reported sporadically since the first reported case by Carlos et al. in 2015 [5]. The diagnosis is challenging considering the atypical presentation of prolonged pruritus prior to the appearance of the typical blistering disease. Here, we present an interesting case of BP while on pembrolizumab, where the final diagnosis was obtained after careful exclusion of other possible etiologies.

## Case presentation

A 66-year-old male with a medical history of type 2 diabetes mellitus, hypertension, and a recent diagnosis of poorly differentiated grade 3 adenocarcinoma of the right upper lobe of the lung was admitted for evaluation of a rash on his trunk and both upper and lower extremities.

**Figure 1:**
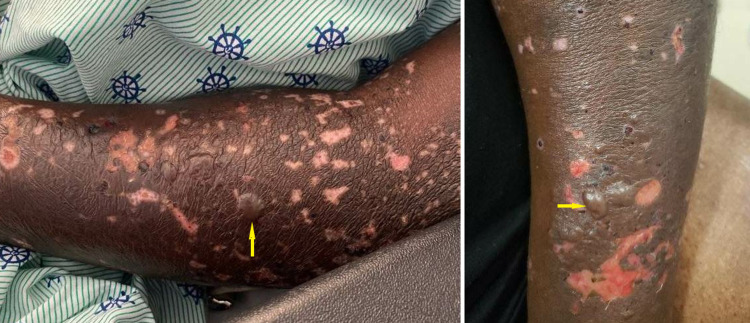
Bullous pemphigoid lesions. Yellow arrows point toward tense bullae among numerous ruptured and partially ruptured bullae on upper extremities.

Approximately one year ago the patient had received the diagnosis of lung adenocarcinoma which showed 90% expression of programmed death ligand-1 (PD-L1). Positron emission tomography at that time demonstrated metastatic lymphadenopathy in the right paratracheal, right perivascular, subcarinal, and right paraesophageal lymph nodes. The adenocarcinoma was staged as T2aN2M1a. Eight months prior to the current presentation, therapy with intravenous pembrolizumab 200 mg every three weeks was initiated. He had completed 21 cycles of pembrolizumab without adverse events. Two weeks prior to current admission, the patient was started on levofloxacin for possible aspiration pneumonia. He reported itching in the left upper arm before levofloxacin was initiated and had pruritic blisters on his left upper extremity in the distribution. The lesions subsequently spread to his left flank. He was diagnosed with shingles in an outpatient setting and prescribed acyclovir. He completed the five-day course of levofloxacin and a seven-day course of acyclovir, but the rash continued to spread to the right upper extremity, trunk, and lower extremities. Despite stopping levofloxacin and completing the course of acyclovir, the rash became more diffuse and pruritic (Figure 1).

**Figure 2:**
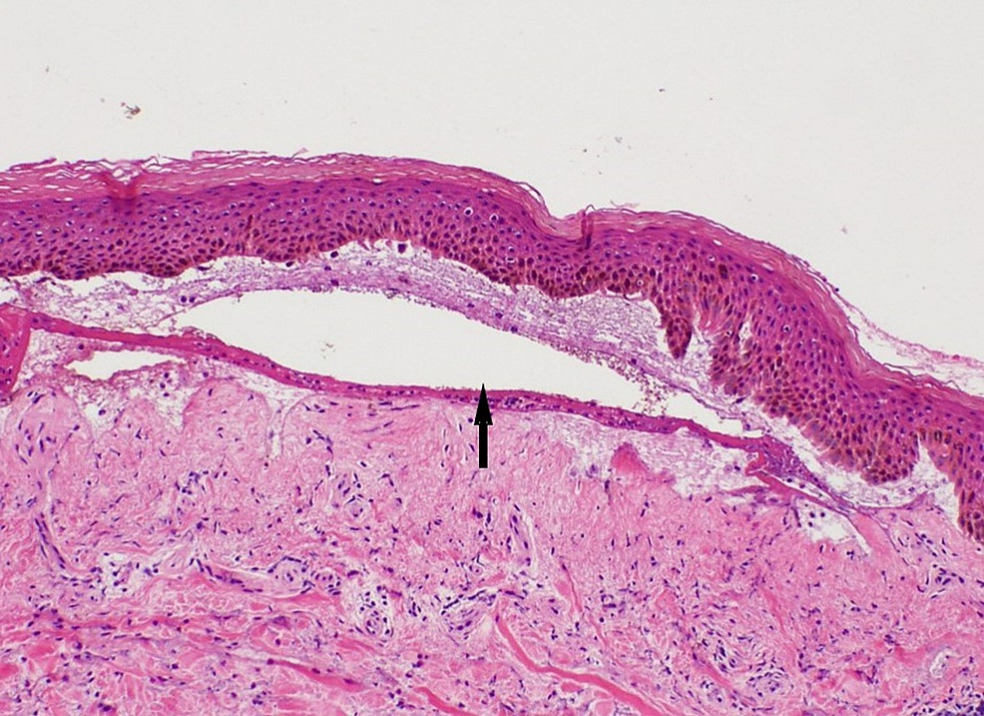
Subepidermal bullous lesion of bullous pemphigoid. Subepidermal vesicle (black upward arrow) with underlying inflammatory infiltrate.

**Figure 3:**
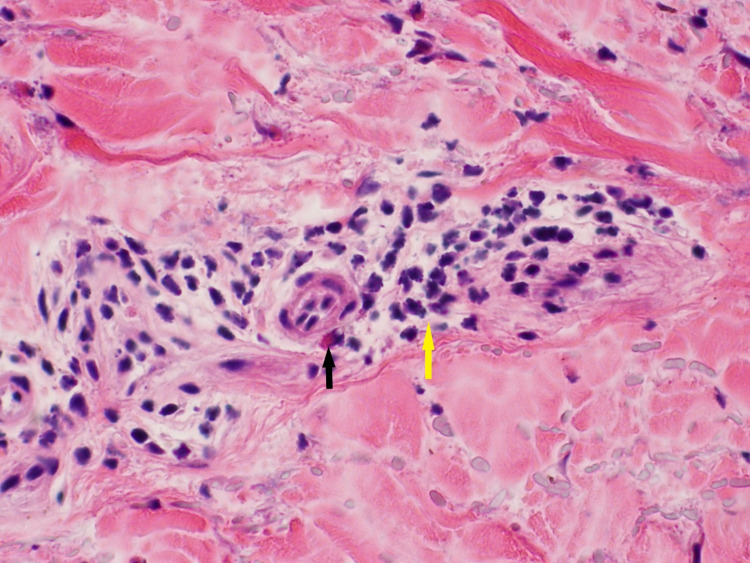
Inflammatory infiltrates within the dermis. Mixed inflammatory infiltrates with superficial perivascular distribution are present in the dermis. The infiltrate is composed of lymphocytes (yellow arrow) with some scattered eosinophils (black arrow) and neutrophils.

At this point, he was admitted to the hospital, and drug-induced BP secondary to pembrolizumab became high on the differential. The patient was afebrile on admission and had stable vitals. Physical examination showed scattered tense bullae on the trunk and both upper and lower extremities with minimal serous drainage from ruptured vesicles. There was no mucosal involvement, and the Nikolsky sign was negative. Pertinent laboratory findings included an eosinophil count of 15.8 10^3^/µL, C-reactive protein 2.25 mg/dL, and an erythrocyte sedimentation rate of 87 mm/hour. Three 5 mm punch biopsies were taken from various sites. Specimen demonstrated subepidermal vesicle formation with several eosinophils (Figures 2, 3).

Direct immunofluorescence of the specimen revealed C3 and immunoglobulin G deposition along the basement membrane. These findings were consistent with drug-induced BP. Pembrolizumab was discontinued, and he was treated with dexamethasone 4 mg twice a day, topical clobetasol, and hydroxyzine 25 mg three times a day with a good response. At the time of writing this report, the patient’s adenocarcinoma was stable, and he was not started on any other chemotherapy by his oncologist.

## Discussion

Pemphigoid disorders are autoimmune bullous dermatoses with autoantibodies against structural components at the dermal-epidermal junction leading to subepidermal splitting which leads to blistering of the skin [1,6]. BP is the most common pemphigoid disorder with autoantibodies against dystonin (implicated autoantigen BP180), which are hemidesmosomal proteins involved in adhesion at the dermal-epidermal junction [1,2]. It mostly affects elderly patients more than 60 years of age [7]. Although the risk factors of pemphigoid disorders are not clear, around 30-50% of cases are reported in patients with dementia, Parkinson’s disease, epilepsy, and a history of stroke [6]. Of note, the autoantigen BP180 is also found in the central nervous system (CNS) and may explain the correlation between CNS disorders and BP [1].

Pembrolizumab is an immune checkpoint inhibitor with expanding indications for the treatment of tumors expressing PD-L1 and PD-L2, which are ligands for the PD-1 receptor on activated T cells. The binding of PD-L1 or PD-L2 to PD-1 leads to the inhibition of the cytotoxic T cell response [8]. Thus, monoclonal antibodies against PD-1 can be used to inhibit this mechanism and allow for cytotoxic T cell recognition of tumor cells in various malignancies. One growing use for pembrolizumab is in non-small-cell lung cancer (NSCLC) because it has been demonstrated that pembrolizumab has greater efficacy and fewer adverse effects than platinum-based chemotherapy for NSCLC with at least 50% expression of PD-L1 [9,10].

Several commonly used drugs can induce or trigger BP including commonly used antibiotics, non-steroidal anti-inflammatory drugs, calcium channel blockers, Angiotensin-converting enzyme inhibitors, angiotensin II antagonists, and beta-blockers [11]. In 2015, Carlos et al. described the first case of pembrolizumab-induced BP in a patient with metastatic melanoma [5]. Schmidt and Zillikens reported cases of checkpoint inhibitor-associated mucous membrane pemphigoid [1]. Drug-induced BP secondary to pembrolizumab can be an extremely challenging diagnosis. In this case, the patient had received levofloxacin, which has a known association with drug-induced BP [11]. However, given that the symptoms of itching and rash started before the initiation of levofloxacin, it is unlikely that this was the cause of the patient’s BP. The second challenge to arriving at this diagnosis is the varying time course for the presentation of BP secondary to pembrolizumab. In a meta-analysis of patients who had developed drug-induced BP secondary to PD-1 and PD-L1 inhibitors, it was demonstrated that patients taking pembrolizumab developed bullae anywhere from 16 to 84 weeks after initiating therapy with a median duration of 28 weeks [11]. Lopez et al. also reported pruritus as the most common symptom of BP in patients on PD/PDL-1 inhibitors and delayed development of blisters in these cases [11]. This patient developed bullae 63 weeks after initiation of treatment. With a variable time to onset of the complication and several commonly used drugs as confounders, it can be extremely difficult to arrive at the diagnosis of drug-induced BP secondary to pembrolizumab.

Treatment of BP secondary to checkpoint inhibitors is the same as treatment for BP secondary to other agents. The drug in question should be discontinued. Patients should be given low-dose oral corticosteroids in conjunction with potent topical corticosteroids [6,12]. Patients usually have complete remission within six weeks of initiation of treatment [13]. Sharma et al. reported a case of pembrolizumab-induced BP treated with rituximab [14]. In cases not responding to corticosteroid and withdrawal of the offending agent, the use of monoclonal antibodies may be considered [15]. In our patient, pembrolizumab was discontinued, and the patient responded well to dexamethasone. Regarding his NSCLC, he is being watched with serial imaging off all immunomodulator therapy.

## Conclusions

With increasing indications for pembrolizumab and other immune checkpoint inhibitors, it is extremely important to be aware of drug-induced BP as a rare complication. As mentioned previously, this can be an extremely challenging diagnosis to make. However, with increased indications, this complication will likely increase in frequency. Physicians should be aware of this potential complication and keep it on their differential diagnosis when treating patients on immune checkpoint inhibitors such as pembrolizumab who develop dermatologic lesions such as BP. Varying times to develop BP implies that it should remain on the differential regardless of how far along the patient is on their treatment course.
